
JOURNAL INFORMATION
==============================
NLM Title Abbreviation: JBRA Assist Reprod
Journal ID: JBRA Assist Reprod
NLM Journal ID: 101684552
Journal ID: jbra

JBRA Assisted Reproduction

ISSN: 1517-5693
EISSN: 1518-0557
Publisher: Brazilian Society of Assisted Reproduction

ARTICLE INFORMATION
==============================
PMCID: PMC6501746
PMID: 31038296
DOI: 10.5935/1518-0557.20190026
Article version: 1
Subjects: Poster Presentations

Abstracts of the 14th Red Lara Taller General, Mérida, Mexico, 06-9 March 2019

Publication date: 2019 Apr-Jun
Print publication date: 2019 Apr-Jun
Volume: 23
Issue: 2
First page: 182
Last page: 196
License: This is an Open Access article distributed under the terms of the Creative Commons Attribution License, which permits unrestricted use, distribution, and reproduction in any medium, provided the original work is properly cited.
License URL: https://creativecommons.org/licenses/by/4.0/

==============================


P-01. The use of hysteroscopy in patients with fertility disorders

Reyes Pérez A 1 2
de Regla Rojas Quintana P 1 2
Chávez González N 1 2
Reyes Pérez AM 1 2
1 "Gustavo Aldereguia Lima" General University Hospital. Cienfuegos. Cuba.
2 Assisted Reproduction Territorial Center. Cienfuegos. Cuba.

P-02. Serum metabolomic profile as a non-invasive tool for the diagnosis of endometriosis-related infertility

Braga DPAF 1 2
Montani DA 3
Setti AS 1 2
Iaconelli Jr. A 1 2
Silva DO 3
Borges Jr E 1 2
1 Fertility Medical Group, São Paulo - Brazil.
2 Instituto Sapientiae - Centro de Estudos e Pesquisa em Reprodução Assistida, São Paulo - Brazil.
3 Instituto de Ciências Ambientais, Químicas e Farmacêuticas - Grupo de Bioorganica e Bioanalítica, Universidade Federal de São Paulo - UNIFESP, São Paulo - Brazil.

P-03. Semen quality difference between samples collected on the day of intra uterine insemination versus at the initial evaluation of male fertility

Puga-Torres T 1
Blum-Rojas X 1
Blum-Narváez M 1
Mera-Intriago E 2
Morey-León G 3
López-Montanero E 4
1 Innaifest Assisted Reproduction Center, Guayaquil-Ecuador.
2 Escuela Superior Politécnica Del Litoral, Guayaquil-Ecuador.
3 University of Guayaquil, Guayaquil-Ecuador.
4 Universidad Espíritu Santo, Samborondón-Ecuador.

P-04. Influence of women age, ovarian stimulation protocol and follicular size on the number of Metaphase II oocytes recovered

Puga-Torres T 1
Blum-Rojas X 1
Blum-Narváez M 1
Mera-Intriago E 2
Romar R 3
Cánovas S 3
1 Innaifest Assisted reproduction center, Guayaquil-Ecuador.
2 Escuela Superior Politécnica del Litoral, Guayaquil-Ecuador.
3 University of Murcia, Murcia-Spain.

P-05. Pursuing the euploid embryo: How many cycles do we need?

Miguens M 1
Coscia A 1
Lorenzi D 2
Bilinski M 2
Madera Espinal M 2
Papier S 1
1 CEGYR, Buenos Aires, Argentina.
2 NOVAGEN, Buenos Aires, Argentina.

P-06. Does oocyte age influences morphokinetics score? Oocyte age, Vitrolife's KIDScore and blastulation timing correlations for clinical pregnancy using a Time-lapse system

Jacobs CK 1
Nicolielo M 1
dos Reis AP 1
Lorenzon AR 1
da Motta ELA 1 2
Alegretti JR 1
1 Huntington Medicina Reprodutiva, São Paulo, SP, Brazil.
2 School of Medicine, Federal University of São Paulo, São Paulo, SP, Brazil.

P-07. Successful delivery of a healthy baby following the transfer of embryos cryopreserved for 17 years

Silva AA 1
Nakagawa HM 1
Amaral MEB 1
Barbosa MWP 1
Barbosa ACP 1
Cabral IO 1
1 GENESIS - Center for Assistance in Human Reproduction, Brasília, DF, Brazil.

P-08. Delay in the blastulation day and the aneuploidy rate

Roble MS 1
Colabianchi M 1
Colabianchi R 1
Fontela S 1
Pérez Audero ME 1
1 Instituto de Fertilidad Asistida "DR. JULIO COLABIANCHI", Rosario, Santa Fe, Argentina.

P-09. Ovarian stimulation in cancer patients: Random versus Conventional start

Coscia A 1
Miguens M 1
Calvo M 1
Anria R 1
Papier S 1
1 CEGYR, Buenos Aires, Argentina.

P-10. Argentinian experience of preimplantation genetic testing for chromosomal structural rearrangements using Microarray-based Comparative Genomic Hybridization and Next-Generation Sequencing

Bilinski M 1
Lorenzi D 1
Menazzi S 1
Quinteiro Retamar A 2
Nodar F 1 2
Papier S 1 2
1 NOVAGEN, Buenos Aires, Argentina.
2 CEGYR, Buenos Aires, Argentina.

P-11. Results from more than 1000 embryos analyzed in-house for preimplantation genetic testing for aneuploidies

Lorenzi D 1
Bilinski M 1
Quinteiro Retamar A 2
Noblía Felicitas 2
Nodar Florencia 1 2
Papier Sergio 1 2
1 NOVAGEN, Buenos Aires, Argentina.
2 CEGYR, Buenos Aires, Argentina.

P-12. Evaluation of oocitary dysmorphism under seasons influence

Saruhashi IT 1
Scaraboto D 1
Cho RY 1
de Lima RA 1
Dal Toso KNF 1
Centa LJR 1
1 ANDROLAB - Clínica e Laboratório de Reprodução Humana Assistida.

P-13. correlation between oocitary dysmorphism and the patient age

Saruhashi IT 1
Scaraboto D 1
Cho RY 1
de Lima RA 1
Dal Toso KNF 1
Centa LJR 1
1 ANDROLAB - Clínica e Laboratório de Reprodução Humana Assistida.

P-14. Standard of seminal analysis in patients with normal and altered BMI from an assisted reproduction clinic

Saruhashi IT 1
Scaraboto D 1
Cho RY 1
de Lima RA 1
Dal Toso KNF 1
Centa LJR 1
1 ANDROLAB - Clínica e Laboratório de Reprodução Humana Assistida.

P-15. Comparison of three expanded carrier screening panels at a single fertility centre

Fabbro M 1
Quinteiro Retamar A 2
De Carvalho P 2
Lorenzi D 1
Bilinski M 1
Menazzi S 1
Galain M 1
Ortiz Maffei N 2
Anria R 2
Nicotra Perassi P 1 2
Nodar F 1 2
Fiszbajn G 2
Fernández C 1
Papier S 1 2
1 NOVAGEN, Buenos Aires, Argentina.
2 CEGYR, Buenos Aires, Argentina.

P-16. Analysis of products of conception by next-generation sequencing

Lorenzi D 1
Bilinski M 1
Nicotra Perassi P 1 2
Menazzi S 1
Artazcoz S 2
Fiszbajn G 1 2
Papier S 1 2
1 NOVAGEN, Buenos Aires, Argentina.
2 CEGYR, Buenos Aires, Argentina.

P-17. Comparison of CFTR mutation frequency using three different expanded carrier screening tests in an egg donation program

Bilinski M 1
Fabbro M 1
Fernández C 1
Menazzi S 1
Quinteiro Retamar A 2
De Carvalho P 2
Ortiz Maffei N 2
Anria R 2
Lorenzi D 1
Galain M 1
Fiszbajn G 2
Papier S 1 2
1 NOVAGEN, Buenos Aires, Argentina.
2 CEGYR, Buenos Aires, Argentina.

P-18. Is the oocyte fertilization affected by maternal age?: An analysis in 47,454 inseminated oocytes

Villanueva P 1 2
Noriega-Hoces L 1 2
Vizcarra EF 1
Llerena G 1 2
Noriega J 1 2
Meza J 1 2
Guzmán L 1 2
1 PRANOR. Grupo de Reproducción Asistida. San Isidro. Lima-Peru.
2 Clínica Concebir. San Isidro. Lima-Peru.

P-19. Associated factors with total fertilization failure after assisted reproductive treatments: Analysis of 14 years

Villanueva P 1 2
Noriega-Hoces L 1 2
Vizcarra F 1
Llerena G 1 2
Inoue N 1 2
Meza J 1 2
Guzmán L 1 2
1 PRANOR. Grupo de Reproducción Asistida. San Isidro. Lima-Peru.
2 Clínica Concebir. San Isidro. Lima-Peru.

P-20. Report of pregnancy success after in vitro fertilization in women aged 40 and over with fresh non donor oocytes in Red Crea, Mexico

Blancas López V 1
Pérez Quijano Y 1
Alvarado Enriquez M 1
Maquita Nakano C 1
Mejia García C 1
Bustamante Mendoza J 1
Flores Islas L 1
1 Red Crea Medicina Reproductiva, CDMX, México.

P-21. Case report of severe male factor in old age and Preimplantation Genetic Diagnosis

Aguilar Mancera V 1
Alvarez Olvera TI 1
Velázquez Maldonado HA 1
de Alba Cervantes MG 1
Carrillo Ruiz Velasco D 1
Quiroz Torres E 1
1 Life CryoBank, Guadalajara, Jal. Mexico.

P-22. PGS contribution to pregnancy rates in different age groups, with own eggs

Rodrigues Hassam D 1
Morales Gómez Y 1
Moreno Barragán C 1
Soto-Rodríguez S 1
Torres Rivera A 1
Gaytán Melicoff J 1
1 Advanced Fertility Center Cancún, Cancún, México.

P-23. Have we reached the peak in vitrification? Improved vitrification method yields to the highest worldwide positive pregnancy outcomes compared to fresh cycles

Soto-Rodríguez S 1
Rodrigues-Hassam D 2
Kuwayama M 1
1 Research Department. Repro-Support Medical Research Centre. Cancún, México.
2 IVF department. Advanced Fertility Center. Cancún, México.

P-24. Occupational Risk - induced sperm DNA damage assessed by comet assay: a pilot study

Zevallos A 1
Rosales J 2
Pella R 1
Romero S 1
1 Centro de Fertilidad y Reproducción Asistida (CEFRA), Lima, Peru.
2 Centro Nacional de Salud Ocupacional y Protección del Medio Ambiente para la Salud (CENSOPAS), Instituto Nacional de Salud (INS), Lima, Peru.

P-25. Assessing embryo quality to predict pregnancy

Abulafia L 1
Kimelman D 1
Cantú L 2
Dellepiane M 1
de la Fuente G 1
1 Department of Gynecology at Centro de Esterilidad Montevideo (CEM). Uruguay.
2 Department of Embryology and IVF laboratory at Centro de Esterilidad Montevideo (CEM). Uruguay.

P-26. hCG as a predictor of pregnancy evolution in patients undergoing IVF

De la Fuente G 1
Decia M 1
Goyeneche L 1
Cantu L 1
1 Centro de Esterilidad Montevideo (CEM). Montevideo. Uruguay.

P-27. Characterization of the Uruguayan semen donor population

Barrera N 1
Ordoqui R 1
Montes JM 1
Canepa M 1
Bonelli C 1
Surka C 1
Torrens A 1
Cantú L 1
Du Plessis SS 2
1 Fertilab Laboratorio de Análisis Clínicos, Laboratorio de Andrología, Montevideo, Uruguay.
2 Medical Physiology, Faculty of Medicine and Health Sciences, Stellenbosch University, Tygerberg, South Africa.

P-28. Oxidative stress and their relationship with semen parameters and reproductive outcome of in vitro fertilization (IVF) cycles with donor eggs: A retrospective study

Barrera N 1 2
Bonelli C 1 2
Surka C 1
Montes JM 1
Ordoqui R 1
Canepa M 1
Torrens A 1
Cantú L 1 2
1 Fertilab, Laboratorio de Análisis Clínicos, Laboratorio de Andrología, Montevideo, Uruguay.
2 Centro Esterilidad Montevideo (CEM), Laboratorio de Fertilización In vitro, Montevideo, Uruguay.

P-29. A retrospective study of egg donation program: comparison outcome of fresh and vitrified donor oocytes

Goyeneche L 1
Barrera N 1
Mas de Ayala J 1
Alciaturi J 1
Bonelli C 1
García R 1
Cantú L 1
1 Centro de Esterilidad Montevideo (CEM), IVF Laboratory, Montevideo, Uruguay.

P-30. Outcome after late rescue Intracytoplasmic Sperm Injection

Alciaturi J 1
Mas de Ayala J 1
Barrera N 1
Goyeneche L 1
Bonelli C 1
García R 1
De la Fuente G 1
Cantú L 1
1 Laboratorio de Fertilización in vitro Centro de Esterilidad Montevideo (CEM), Montevideo, Uruguay.

Soledad Sepulveda Award

Best Poster Presentation

14th REDLARA General Congress, Merida, Mexico - 2019

